# Treatment of Rheumatism

**Published:** 1844-02-24

**Authors:** 


TREATMENT OF RHEUMATISM.

Two different remedies are highly praised, at the present moment, by some of the French physicians, in the treatment of this very common disease. These are, the sulphate of quinine and the nitrate of potash. M. Briquet, and a host of followers, descant, in terms of no measured commendation, on the virtues of quinine; while M. Martin Solon is no less energetic in his laudations of his favourite. Neither of these gentlemen seems to be at all imbued with homœopathic principles, as far at least as regards the doses which they administer; for the one is in the habit of giving from 15 to 20 grains of quinine, and the other from half an ounce to an ounce and a half of the nitrate, at a time. We need scarcely say that we utterly disapprove of the indiscriminate practice of both.

Our chief motive for alluding to this subject at present, is to introduce the following sensible remarks by a writer in the Gazette Medicale, in reference to Dr. Solon's Memoir. "No one has ever questioned the perfect accuracy of the assertions made by this gentleman. As far as we can judge, we should say that the nitrate of potash, administered in large doses, is not only inoffensive, but is often very useful. There is, however, in all the statements and reports that have been made on the subject, an important blank, which we should have wished to have seen filled up. Be it remembered, that not a few cases of rheumatism have a natural tendency to relieve themselves by perspiration, if the fever do not run high, and the pains are not very severe; whereas, the disease is apt to be obstinate and unyielding, when the symptoms are otherwise. It certainly does seem to us that many of the cases related by Dr. Solon, might have got well if nothing had been given except simple warm drinks.

"This remark applies with equal force to every other remedy that has, at different times, been recommended for the cure of rheumatism, as well as to the nitrate of potash. It requires, therefore, much more discrimination than is usually imagined, to determine with accuracy the genuine therapeutic effects of medicines."

Perfectly true. The credit, that properly belongs to the *vis medicatrix naturæ*, is not unfrequently awarded to the medicines that are administered.—*Lon. Medico-Chirurgical Review.*

